# A Biomechanical Evaluation of Distal Tilting Implants in All-on-Four Rehabilitation with Mild Mandibular Resorption: A Finite Element Analysis Study

**DOI:** 10.3390/ma17225435

**Published:** 2024-11-07

**Authors:** Ming-Hsu Tsai, Chung-Han Lee, Aaron Yu-Jen Wu, Yao-Ning Lei, Hung-Shyong Chen, Yu-Ling Wu

**Affiliations:** 1 Department of Mechanical Engineering, Cheng Shiu University, Kaohsiung 833, Taiwan; 2 Department of Dentistry, Kaohsiung Chang Gung Memorial Hospital, College of Medicine, Chang Gung University, Kaohsiung 833, Taiwan; 3 Kaohsiung Municipal Feng Shan Hospital—Under the Management of Chang Gung Medical Foundation, Kaohsiung 830, Taiwan; 4 Department of Dentistry, Chang Gung Memorial Hospital, Linkou, Chang Gung University, Taoyuan City 333, Taiwan

**Keywords:** implant–abutment connections, all-on-4 treatment, finite element analysis

## Abstract

The geometry of implants plays a crucial role in the success of All-on-Four treatments for the lower jaw. This study builds upon prior research by evaluating the biomechanical performance of implant-supported prostheses in full-arch fixed dental restorations, specifically focusing on different implant lengths and connection types in cases of mild atrophic resorption of the mandible. Four groups were analyzed using finite element analysis (FEA): We utilized 13 or 18 mm posterior 17-degree tilting implants, each paired with two kinds of abutment connections. The external hexagon connection (EHC) group utilized 4 mm diameter implants, while the internal hexagon connection (IHC) group employed 4.3 mm diameter implants. A vertical force was applied to the cantilever region located at the distal side of the posterior implant. The maximum stress regions were observed in prosthetic screws and multi-unit abutments (MUAs) across all groups, with the lowest von Mises stress values noted in the bone. Stress peaks for implant screws and fixtures in the 13 mm group were 19.98% and 11.42% lower, respectively, compared to the IHC group. Similarly, in the 18 mm group, stress peaks were reduced by 33.16% and 39.70% for the EHC group compared to the IHC group. The stress levels on all components remained below the ultimate strength of the titanium alloy. For the same implant lengths, the stress in the prosthetic screw, MUAs, implant screw, and implant fixture positions was lower in the EHC group. When implant length was increased, a decrease in stress levels was observed in the implant screw and fixture of the EHC group and only in the implant screw of the IHC group. However, an increase in stress was noted in the prosthetic screw and MUAs for both groups.

## 1. Introduction

In clinical practice, the selection of implant length for a single implant is often influenced by bone height and the need to increase the implant’s surface area. Increasing the length of the implant can enhance the surface area, thereby improving its primary stability [1]. Systematic reviews indicate that longer implants are beneficial when feasible [2,3].

For the reconstruction of edentulous patients, the “All-on-Four” treatment, introduced by Maló et al., is a widely used option [4]. This technique entails the vertical placement of two anterior implants and the angulated placement of two implants in the posterior region, thereby providing support for a full-arch fixed prosthesis and promoting the efficient distribution of occlusal forces. Dr. Maló defined and discussed implant lengths in the context of All-on-Four treatment, with its success rates reported in several systematic reviews [5].

When placing posterior implants in an All-on-Four treatment, various angles, lengths, and connection types must be considered [6]. According to existing studies and descriptions of the surgical procedure, posterior implant tilting may range from 15° to 45° [7,8]. The choice of tilt is influenced by several factors, including avoidance of the inferior alveolar nerve, bypassing the mental foramen, ensuring initial stability by engaging the lateral cortical bone, and achieving better anterior–posterior spread. Pelekhan et al. also developed a mechanical and mathematical model for the All-on-Four system to examine the stress conditions experienced by implants and the adjacent bone tissue when subjected to masticatory forces. This method allows for the final assessment of the strength of the implants and bones through calculations, providing design recommendations for the positioning and sizing of the implants [9]. In clinical situations, particularly when the mandibular bone is high, as seen in mildly atrophic mandibles due to trauma or a high rate of caries [10], the anterior loop may not be inclined. Surgeons must ensure a safe zone during surgery, which may lead them to opt for a less pronounced tilt of the implant. These decisions can also impact the future fabrication of prostheses [11].

Complications associated with All-on-Four treatment are closely linked to implant selection and prosthesis design. Complications such as fractures of prosthetic devices and the loosening of implant or prosthetic screws (P-screws) can significantly impact the overall efficacy of treatment outcomes [5,6]. These complications are predominantly related to occlusal overload, where excessive occlusal forces may contribute to marginal bone loss. Differences in implant design, including aspects such as length, angulation, and the nature of the implant–abutment connection, play a significant role in influencing the distribution of occlusal forces [12]. Variations in design can have a substantial impact on the performance and maintenance of implant osseointegration, which may result in both mechanical and biological complications [13]. These issues may jeopardize treatment outcomes and could escalate to more severe problems, including implant fractures [14].

Implant–abutment interface designs can be categorized into two types, external and internal hexagon connections, based on the various types of implant–abutment configurations. In cases of single implants, internal connections have become the standard, supported by literature indicating less marginal bone loss [15,16]. The connection type is also a key consideration in All-on-Four treatments. Although systematic reviews do not conclusively favor a specific connection type, studies suggest no significant difference in outcomes between internal hexagon connections (IHC) and external hexagon connections (EHC) [17]. Dr. Maló has predominantly utilized EHC [4,6]; however, there is limited detailed research comparing these differences.

Research has examined the impact of posterior implant length on the surrounding bone in All-on-Four treatments, recommending the use of longer implants in the posterior area [18]. Several research investigations have examined the impact of cantilever length and the placement of posterior implants [8,19]. However, these studies often overlook the effects on prosthetic components and the types of implant connections used. For All-on-Four treatments, prosthetic complications are a critical issue for long-term maintenance.

Therefore, this study builds upon previous research by incorporating length considerations as model conditions [20], and specifically focuses on investigating the biomechanical behavior of the components in All-on-Four treatments under different implant–abutment connection types and varying lengths. The aim is to provide insights into how implant length and implant–abutment connection types affect clinical outcomes, with the goal of improving the clinical success of All-on-Four treatments.

## 2. Materials and Methods

### 2.1. Three-Dimensional FEA Modeling

This research examined a particular model representing the All-on-Four treatment for mildly resorbed mandibles, including two anterior implants and two posterior implants. The components of the All-on-Four comprised a tailor-made titanium implant bar, multi-unit abutments (MUAs), P-screws, and implant screws (I-screws). The anterior implants had a diameter of 4 mm and a length of 13 mm (NobelSpeedTM Groovy, Nobel Biocare, Goteborg, Sweden) with 1 mm straight MUAs (Multi-unit Abutment, Nobel Biocare, Goteborg, Sweden). For the posterior implants, two connection types with different lengths and the same tilting angle (17°) were employed: EHC with 4 mm diameter implants of 13- and 18 mm lengths (NobelSpeed^TM^ Groovy, Nobel Biocare) and IHC with 4.3 mm diameter implants of 13 and 18 mm lengths (NobelParallel^TM^ Conical Connection, Nobel Biocare). Both groups used 17° MUAs (Nobel Biocare), with the height of the MUAs being 2.5 mm in the IHC group and 2 mm in the EHC group.

**Figure 1:**
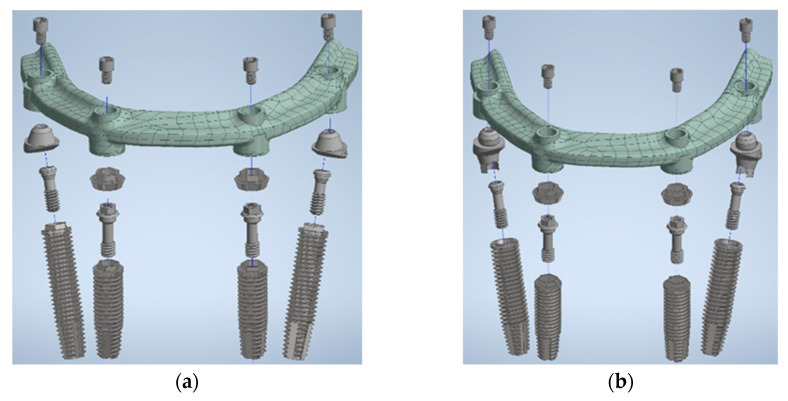
Finite element model in (**a**) the IHC group and (**b**) the EHC group.

All measurements of components were conducted utilizing a digital microscope and vernier calipers to guarantee accuracy. Detailed images were captured through a high-resolution 3D optical scanning system (Aicon SmartScan-HE by Breuckmann, Braunschweig, Germany). Subsequently, three-dimensional models were generated employing computer-aided design (CAD) software (Inventor 2020 by Autodesk, San Rafael, CA, USA) and FEA software (ANSYS Workbench 2020 R1 by ANSYS, Inc., Canonsburg, PA, USA). The FEA models of the All-on-Four assemblies for the experimental groups are illustrated in Figure 1.

In ANSYS Workbench 2020 R1, the model under analysis was integrated into a bone block model measuring 5 cm × 3 cm × 4 cm to replicate human bone structure. This model included a 0.3 cm thick outer layer designed to mimic cortical bone and an inner layer representing cancellous bone with a spongy structure.

### 2.2. Three-Dimensional FEA Model Analysis

The tetrahedral elements (SOLID187) were utilized for meshing all components in the study, demonstrating quadratic displacement behavior. Element sizes ranged from 0.08 to 2.00 mm to ensure precise results. All groups were comparable in terms of the number of elements: the external hexagon connection group consisted of 1,885,434 elements and 2,842,741 nodes, while the internal hexagon connection group comprised 1,948,198 elements and 2,954,778 nodes.

The components investigated in the research were validated using energy-dispersive X-ray spectroscopy (JSM-6360; JEOL, Tokyo, Japan). The mechanical characteristics of these materials have been elaborated upon in a prior study [21,22,23]. The detailed values are enumerated in Table 1.

The interface bonding between cortical and cancellous bone was modeled to emphasize the loading effects on the components. There would be complete osseointegration between the implants and the bordering bone. Frictional effects within the All-on-Four model were modeled using a coefficient of 0.3 [24].

**Figure 2:**
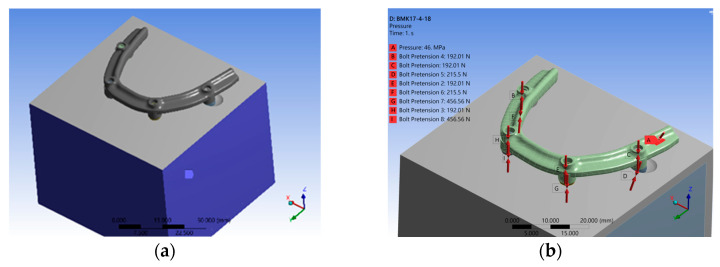
The model utilized in this research was subjected to specific boundary and loading conditions. (**a**) With the exception of the occlusal surface, the bone block model was firmly stabilized on all surfaces. (**b**) A vertical force of 190 N was applied on the cantilever region located 10 mm away from the distal side of the posterior implant, denoted by arrow A. Additionally, bolt pretension was applied to the P-screws and I-screws, as indicated by arrows B-I.

Regarding boundary conditions, fixed support was applied to all surfaces of the bone block model, limiting displacement in three directions except at the occlusal surface (Figure 2a). The application of force involved converting screw tightening torque to axial force using the formula (T = KDF), where (T) represents tightening torque (N·m), (K) is the torque coefficient, (D) denotes screw diameter (mm), and (F) indicates axial force (N). Recommended torques were 0.1 N·m for P-screws, 0.35 N·m for the mesial I-screw surface, and 0.15 N·m for the distal I-screw surface, resulting in axial forces of 192.01 N, 457.56 N, and 215.51 N, respectively. A vertical force of 190 N was applied to the cantilever region located 10 mm away from the distal side of the posterior implant (Figure 2b).

## 3. Results

Maximum stress values were observed in the P-screws and MUAs, indicating these as potential weak points in the All-on-Four system. However, the maximum stress values for these components were below the yield strength of the titanium alloy (795 MPa), suggesting clinical safety. Table 2 presented the peak values of the von Mises stress in the four groups.

**Figure 3:**
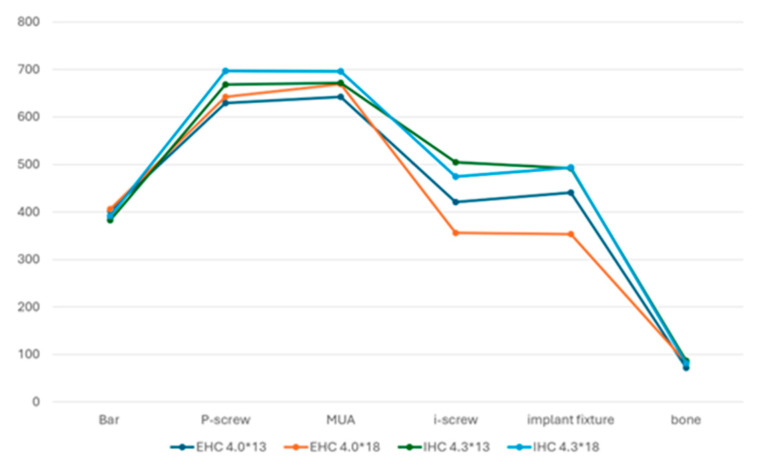
Peak stress at each component across all groups.

When comparing IHC and EHC, the maximum stress values in P-screws, MUAs, I-screws, and fixtures were higher in the IHC system than in the EHC system (Figure 3).

**Figure 4:**
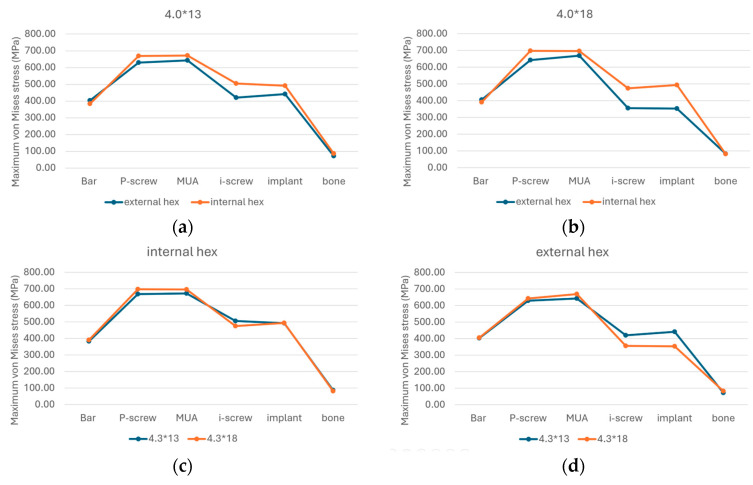
The maximum stress experienced by each component of (**a**) the 13 mm group, (**b**) the 18 mm group, (**c**) the IHC group, and (**d**) the EHC group.

In the 13 mm group, the EHC system demonstrated stress reductions of 6.17% in the P-screw, 4.54% in the MUA, 19.98% in the I-screw, 11.42% in the implant fixture, and 19.69% in the bone compared to the IHC system. Conversely, the stress in the implant bar increased by 4.91% (Figure 4a).

In the 18 mm group, the EHC system showed stress reductions of 8.45% in the P-screw, 4% in the MUA, 33.16% in the I-screw, and 39.70% in the implant fixture compared to the IHC system, while the stress in the implant bar and bone increased by 3.61% and 2.67%, respectively (Figure 4b).

For the IHC system, increasing implant length resulted in increased stress in the bar, P-screw, and MUA by 2.05%, 4.09%, and 3.46%, respectively, when moving from 13 mm to 18 mm, while stress in the I-screw, implant fixture, and bone decreased by 6.43%, 0.42%, and 6.31%, respectively (Figure 4c). Therefore, the choice between these two lengths had a minimal impact on the structure. In the EHC system, increasing length raised stress in the bar, P-screw, MUA, and bone by 0.71%, 2.03%, 3.97%, and 13.56%, respectively, while reducing stress in the I-screw and implant fixture by 18.12% and 24.84%, respectively (Figure 4d), showing a notable improvement in the overall stress distribution.

**Figure 5:**
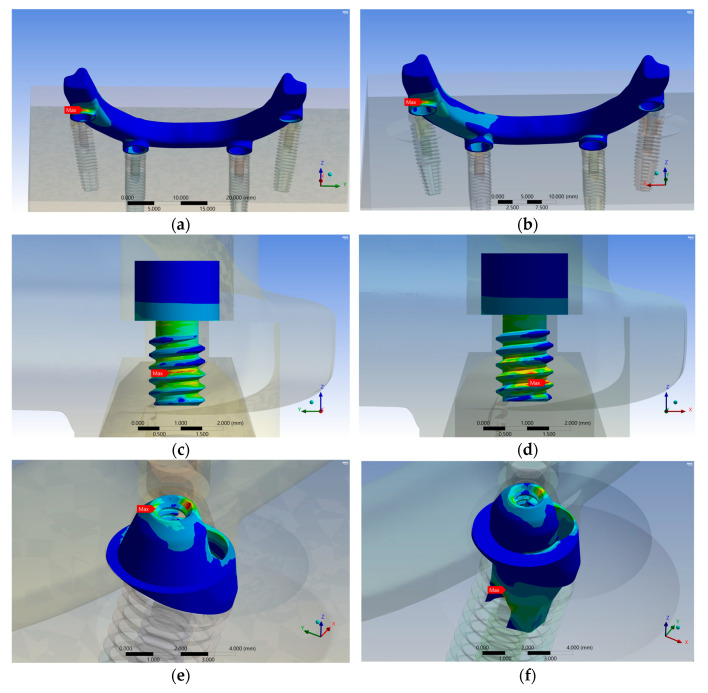

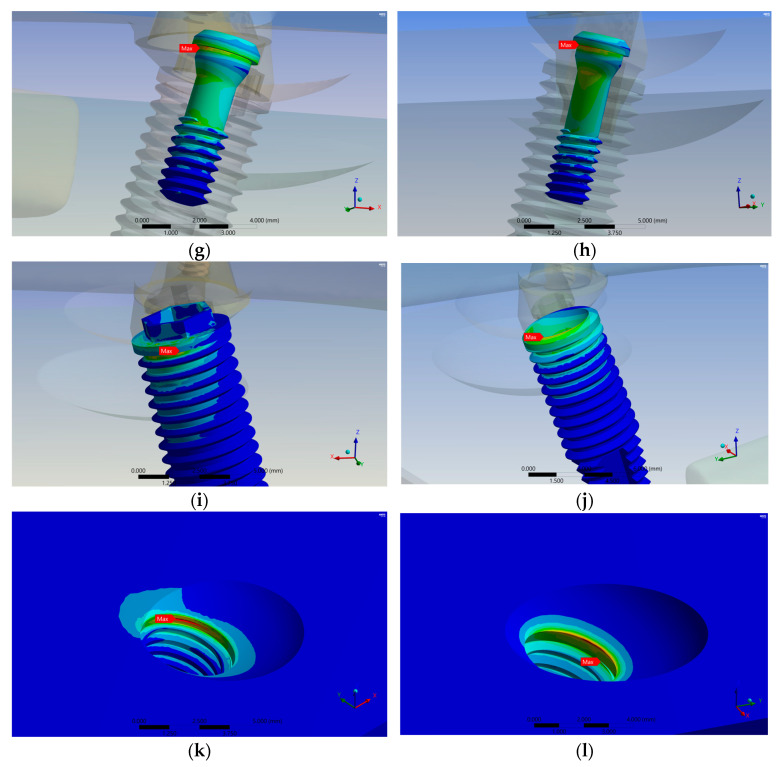
The locations of the maximum von Mises stress and distribution patterns were analyzed for various components in two distinct groups: the 13 mm EHC group and the IHC group. These components included the implant bar (**a**,**b**), P-screws (**c**,**d**), MUA (**e**,**f**), I-screws (**g**,**h**), implant fixtures (**i**,**j**), and bones (**k**,**l**) within each group.

**Figure 6:**
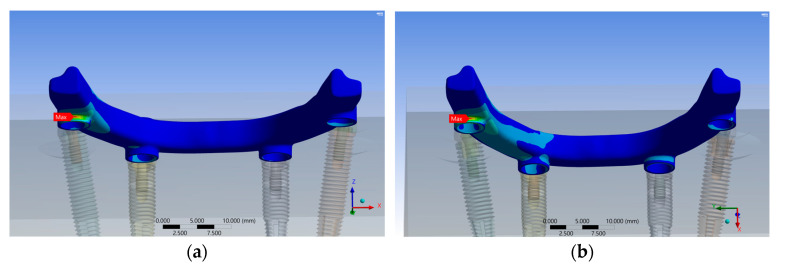

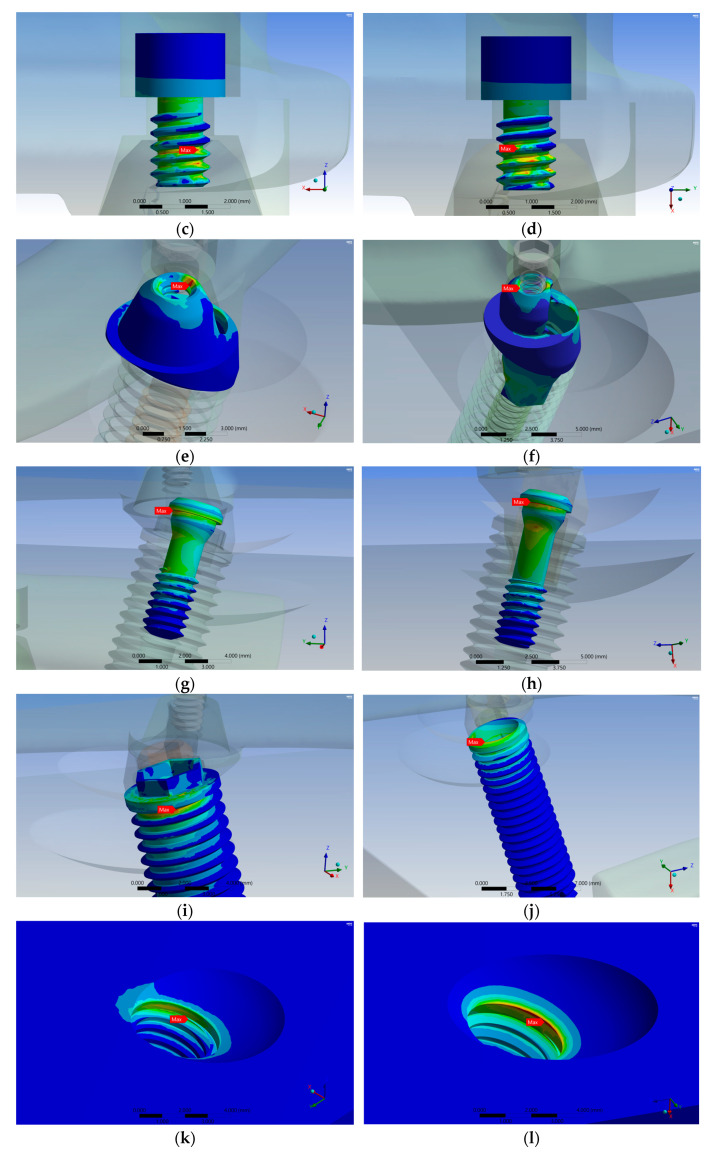
The locations of the maximum von Mises stress and distribution patterns were analyzed for various components in two distinct groups: the 18 mm EHC group and the IHC group. These components included the implant bar (**a**,**b**), P-screws (**c**,**d**), MUA (**e**,**f**), I-screws (**g**,**h**), implant fixtures (**i**,**j**), and bones (**k**,**l**) within each group.

The analysis of load transmission revealed some common stress distribution patterns. Stress tended to accumulate at the interconnections of components. At the distal-lingual surface of the implant bar (Figure 5a,b and Figure 6a,b), the first thread of the I-screw connected to the MUAs (Figure 5g,h and Figure 6g,h), and the cervical third of the implant fixture (Figure 5i,j and Figure 6i,j) exhibited similar stress distribution over all groups. Nonetheless, different areas exhibited varied stress distribution patterns.

In the 13 mm group, the P-screw’s highest stress in the EHC group was observed on the third thread (Figure 5c), while in the IHC group, it was on the fourth thread (Figure 5d). For the MUAs, the highest stress in the EHC group was found at the mesial thread region connected to the P-screw (Figure 5e); nevertheless, it was at the connection with the implant fixture in the IHC group (Figure 5f).

In the 18 mm group, the P-screw’s highest stress in the EHC group was observed on the third thread (Figure 6c), while in the IHC group, it was on the second thread (Figure 6d). For the MUAs, the highest stress in the EHC group was found at the distal thread region connected to the P-screw (Figure 6e); nevertheless, it was at the mesial thread region in the IHC group (Figure 6d).

## 4. Discussion

Selecting the appropriate implant angle and length is crucial in All-on-Four treatment for the lower jaw. This study used a 17-degree angle for the posterior implants. In clinical scenarios with a greater volume of residual alveolar bone, particularly in mild resorption, the amount of bone above the mental foramen is also increased. Consequently, when the implant is positioned in the second premolar or first molar area, the tilting angle is reduced [25]. This is often seen in patients who are edentulous due to trauma or a high rate of caries and require full-mouth rehabilitation. Additionally, when the anterior loop is less inclined, the surgeon must ensure a safe zone during surgery, which may also dictate the tilting angle.

This study builds on previous research exploring the biomechanical effects of implant length and connection type [20]. The effectiveness of load transmission in the All-on-Four treatment is influenced by multiple factors, affecting bone remodeling and potentially leading to implant failure. Such failures can arise from primary factors, including osseointegration failure, or secondary factors, such as marginal bone loss, influenced by local, systemic, surgical, and prosthetic variables [26]. While the implant–abutment connection system may not directly cause failure, it significantly impacts mechanical and biological complications. Additionally, in edentulous patients with implant-supported dentures, chewing forces can stress distal implants and the surrounding bone [27].

FEA studies help elucidate load transmission and stress distribution, improving the evaluation of multi-implant prosthetic treatments, including All-on-Four [28]. This study used FEA to assess load transfer in the posterior implant region of four full-arch implant assemblies. The stress distribution patterns varied between EHC and IHC groups; EHC concentrated stress on denture screws and MUAs, while IHC distributed stress more evenly across implant abutments, screws, MUAs, and bone.

These results align with previous studies, highlighting the superior stress distribution performance of internal connections and their potential to reduce marginal bone loss [13,29,30]. Despite ongoing debates regarding the superiority of connection types, this study identified differences in stress distribution among P-screws, MUAs, I-screws, implant fixtures, and bone across the four groups. The IHC group demonstrated a more uniform stress distribution, but when the length was fixed, the maximum von Mises stress values in the P-screw, MUAs, I-screw, and implant fixture were higher than in the EHC group.

The stress patterns of the EHC and IHC systems at 18 mm were similar to those reported in previous studies, showing that a 17-degree angle reduced stress in the I-screw and implant fixture by 33.16% and 39.70%, respectively, compared to 37.75% and 33.03% at a 30-degree angle [20]. This decrease can be ascribed to the differing stress distribution patterns observed between the MUAs and I-screws within the EHC group, wherein stress is concentrated at the interface between the MUAs and the implant screw. Conversely, stress distribution in the IHC group may increase mechanical complication risks in the lower part of MUAs, a finding noted in several studies [13,29,30]. According to a systemic review, the use of IHC in cases with a single implant can reduce marginal bone loss due to a mismatched platform [16]. Additionally, a separate study indicated that the reduced stress experienced in the bone due to IHC might be attributed to the sliding mechanism present in the joint between the implant and the abutment, which serves to mitigate the effects of bending [31].

Irrespective of the implant–abutment system utilized, the maximum stress values observed were on MUAs, followed by P-screws. In the IHC 18 mm group, P-screws, followed by MUAs, experienced the highest stress values, indicating susceptibility to stress under vertical forces applied to the distal cantilever region of mandibular four-implant assemblies. These components exhibit yield strength values that are comparable to those of titanium alloy (Ti-6Al-4V), which raises apprehensions regarding potential mechanical complications, including fractures of the P-screw and MUAs. The findings align with previous research indicating primary stress concentration on P-screws and MUAs, irrespective of connection type [32,33].

In cases involving single implants, increasing the length enhances the contact area, thereby improving the primary stability of the implant [34]. In All-on-Four treatments, several studies have demonstrated that lengthening tilting implants reduces stress on the crestal bone and surrounding supportive tissues under occlusal loads. This study further investigated the impact of increased length on the prosthetic components. Our findings revealed that lengthening both the IHC and EHC systems resulted in increased stress in the bar, P-screw, and MUAs while reducing stress in the I-screw and fixture. Notably, increasing the length did not reduce the stress at the maximum stress points (P-screw and MUAs) in either group; rather, it increased stress at these points. The EHC system exhibited a greater reduction in stress at the I-screw and fixture compared to the IHC system when the length was increased. In clinical situations, prosthetic complications involving P-screws and MUAs may be easier to manage than those related to I-screws and fixtures. Therefore, we view this stress distribution positively: increasing implant length reduces stress in the lower part of the prosthesis, potentially resulting in fewer complications in that area, while more complications may arise in the upper components. Ultimately, if an I-screw or fixture were to fracture, the repair process could be more time-consuming, and in some cases, a new implant may need to be inserted [35].

Stress arises from the internal restoring force after structural deformation. Maximum stress typically occurs in areas of lower stiffness, with its magnitude influenced by Young’s modulus and the external force applied. In structures with complex shapes, the amount of deformation caused by external forces varies depending on the geometry of the structure. Following the increase in implant length from 13 mm to 18 mm, our analysis indicated a significant reduction in stress on the EHC implant and screw, while stress on the abutment and screw slightly increased. This suggests changes in geometry, rendering the upper abutment area a region of relatively lower stiffness, resulting in increased deformation and stress under load. For the IHC system, the analysis indicated that the maximum stress difference between the 18 mm and 13 mm lengths was minimal, suggesting that lengthening the implant did not significantly enhance structural strength at that diameter. Additionally, the reduction in relative stiffness in the abutment and screw resulted in a slight increase in maximum stress.

The literature review conducted revealed that multiple factors affect load transmission [28]. To specifically examine the influence of connection types, we focused on implants with comparable specifications. A range of parameters was evaluated to ensure the reliability of FEA and the precise modeling of stress distributions. These parameters included assumptions related to the properties of bone tissue, the integration of bone, and the torques applied during screw tightening. Gibbs et al. [36] reported that the average masticatory force exerted by dentate patients is approximately 40% of their maximum biting force during activities. Another study indicated that the maximum biting force in the molar region was measured around 510 N to 550 N for males and about 450 N for females [37]. The research also noted that the force required for food penetration varies according to the type of food [38]. According to the above research, we implemented a vertical force of 190 N in our study, which aligns with 40% of the average maximum biting force.

This research is subject to several limitations. Firstly, the FEA simplified the complexity of human bone, potentially resulting in discrepancies between the analysis results and clinical situations. In our study, we operated under the assumption that bone tissue is isotropic, homogeneous, and exhibits linear elasticity. While these assumptions facilitated the modeling process, they may not have adequately represented the intricate nature of actual bone tissue. Several investigations have utilized linear elastic orthotropic models to enhance the accuracy of predictions related to the mechanical properties of bone tissue [39,40]. Additionally, our study simplified chewing patterns by applying vertical pressure at the distal free end, which does not fully replicate real conditions. Clinically, due to the cusps of prostheses and mandibular movements, prostheses often endure complex biomechanical stresses. Future research should consider applying forces from different directions to more accurately reflect intraoral conditions.

Despite these limitations, FEA offers valuable insights for clinical All-on-Four treatments. The biomechanical results of this study can guide the selection of posterior implants, assisting in the choice of connection systems and implant lengths that minimize stress in the connection area related to prosthetic complications. Future research should include more detailed simulations and validations through clinical trials.

## 5. Conclusions

In light of the findings from this finite element analysis (FEA) investigation, the subsequent conclusions may be articulated, taking into account the constraints of the study:

1. In the 13 mm group, the EHC system demonstrated stress reductions of 19.98% in the I-screw and 11.42% in the implant fixture compared to the IHC system. In the 18 mm group, the EHC system showed stress reductions of 33.16% in the I-screw and 39.70% in the implant fixture compared to the IHC system.
2. Increasing the implant length in the EHC system resulted in greater reductions in maximum stress on the I-screw and fixture. When moving from 13 mm to 18 mm in the EHC system, the maximum stress decreased by 15.34% in the I-screw and 19.90% in the fixture, while in the IHC system, it reduced by 6.43% in the I-screw but increased by 0.42% in the fixture.
3. In both the IHC and EHC groups, increasing implant length led to increased stress at the MUAs and P-screw positions, which already experienced high stress levels.
4. The maximum stress in both systems occurred at the MUAs and P-screw, indicating these components are the weak points in the overall system.
5. The maximum stress values at all locations remained within clinically safe limits.

## Figures and Tables

**Table 1:** Mechanical characteristics of the materials employed in the three-dimensional finite element model.

Materials	Cortical Bone	Cancellous Bone	Titanium (CP Ti)	Ti-6Al-4V (Ti Alloy) Implant Bar, P-Screws, I-Screws, Abutment
Young’s modulus (GPa)	13.4	13.7	115	110
Poisson’s ratio	0.3	0.3	0.35	0.31
Yield strength (MPa)	-	-	680	795

**Table 2:** Maximum von Mises stress values for each component across all groups.

	Component	Implant Bar	P-Screw	MUA	I-Screw	Implant Fixture	Bone
Max Stress							
IHC 13 mm		383.16	668.56	671.89	504.71	491.62	86.55
IHC 18 mm		391.18	697.09	696.02	474.20	493.71	81.41
EHC 13 mm		402.96	629.69	642.69	420.64	441.20	72.31
EHC 18 mm		405.85	642.75	669.26	356.11	353.39	83.65

Unit: MPa.

## Data Availability

The original contributions presented in this study are included in the article. Further inquiries can be directed to the corresponding author.
